# The effects of a single-dose thermogenic supplement on resting metabolic rate and hemodynamic variables in healthy females - a randomized, double-blind, placebo-controlled, cross-over trial

**DOI:** 10.1186/s12970-016-0123-1

**Published:** 2016-03-31

**Authors:** Bill I. Campbell, Gina Zito, Ryan Colquhoun, Nic Martinez, Kristina Kendall, Laura Buchanan, Matt Lehn, Mallory Johnson, Courtney St. Louis, Yasmin Smith, Brad Cloer, Allison Pingel

**Affiliations:** Exercise Science Program, Performance & Physique Enhancement Laboratory, College of Education, University of South Florida, Tampa, FL 33620 USA; Bodybuilding.com, Boise, ID 83713 USA

**Keywords:** Sports nutrition, Metabolism, Dietary supplement, Female physique enhancement, Weight loss, Fat loss

## Abstract

**Background:**

Recent investigations have identified that commercially available dietary supplements, containing a combination of thermogenic ingredients, can increase resting metabolic rate (RMR). Thermogenic dietary supplements can have a positive influence on RMR, but the magnitude can vary based on the active ingredient and/or combination of active ingredients. Additionally, further safety evaluation is needed on multi-ingredient supplements that contain caffeine, due to its potential effect on heart rate (HR) and blood pressure (BP). The purpose of this study was to examine the effects of a commercially available dietary supplement on RMR and hemodynamic variables in healthy females.

**Methods:**

13 female participants (26.1 ± 11.3 years; 163.4 ± 9.1 cm; 63.7 ± 8.0 kg, and 24 ± 5 BMI) volunteered to participate in this investigation. Participants underwent two testing sessions separated by approximately 7 days. On their first visit, participants arrived to the laboratory after an overnight fast and underwent a baseline RMR, HR, and BP assessment. Next, each participant ingested a thermogenic dietary supplement or placebo and repeated the RMR, HR, and BP assessments at 60, 120, and 180-minutes post-ingestion. Approximately 1-week later, the alternative supplement was ingested and the assessments were repeated in the exact same manner. Data were analyzed via a 2-factor [2x4] within-subjects repeated measures analysis of variance (ANOVA). Post-hoc tests were analyzed via paired samples t-tests.

**Results:**

Repeated measures ANOVA revealed a significant effect for time relative to raw RMR data. Post-hoc analysis revealed that the dietary supplement treatment significantly increased RMR at 60-minutes, 120-minutes, and 180-minutes post ingestion (*p* < 0.05) as compared to baseline RMR values. No changes in RMR were observed for the placebo treatment (*p* > 0.05). Heart rate was not significantly affected at any time point with either supplement; however, main effects of treatment and time were observed for both systolic and diastolic blood pressure (*p* < 0.05).

**Conclusions:**

The thermogenic dietary supplement treatment experienced greater elevations in RMR as compared to baseline. Due to the slight elevations in blood pressure, caution should be taken for those with increased risk for hypertension or pre-hypertension. Taken on a daily basis, thermogenic dietary supplementation may increase overall energy expenditure, potentially leading to reductions in fat mass over time.

## Background

Dietary supplements containing thermogenic ingredients are commonly used amongst fitness-minded individuals in an attempt to increase resting metabolic rate (RMR) and facilitate fat loss. Recent investigations have identified that commercially available thermogenic supplements and ingredients typically contained in such products can increase RMR in healthy subjects [1–3], and when taken chronically may elicit positive changes in body composition [4, 5].

Caffeine supplementation has previously been shown to enhance lipolysis and fat oxidation [6]. However, when combined with additional herbal ingredients, the combination appears to be more effective for increasing RMR. [7] The majority of thermogenic supplements contain a combination of dietary ingredients such as caffeine, green tea extract, and various herbal extracts that have been shown to increase metabolism [6–9], decrease body fat [4, 5], and increase markers of lipolysis [10, 11]. Additionally, thermogenic supplements containing caffeine, garcinia cambogia, and chromium polynicotinate have also been shown to increase caloric expenditure [12].

It is generally accepted that caffeine increases resting energy expenditure (REE) through activation of β2 and β3 adrenergic receptors, as well as activation of cyclic AMP (cAMP) [7, 13], causing subsequent increases in circulating epinephrine and free fatty acids [14, 15]. Green tea extract (GTE), which contains high amounts of catechin polyphenols, is also found in many thermogenic supplements and has been shown to increase both energy expenditure and fat oxidation [7, 16–19]. Catechin polyphenols, like epigallocatechin gallate (EGCG), have been found to produce a sparing effect on noradrenaline, ultimately leading to increased levels of the catecholamine which helps to stimulate cAMP [20]. These two ingredients together have been shown to increase REE beyond the individual capabilities of caffeine or GTE [7, 17]. Garcinia cambogia, which comes from a fruit native to India, has been suggested to be a natural weight loss aid. Its primary extract, a substance called hydroxycitric acid (HCA), has been shown in animal studies to block the conversion of sugars to fat [21, 22]. In humans, some evidence suggests it can enhance weight loss [23, 24], while other investigations have reported it is ineffective for inducing weight loss in adults [25]. Yerba mate, commonly consumed as an herbal tea beverage, can also be found in dietary supplements marketed at weight loss. Yerba mate has central nervous system-stimulant properties similar to caffeine, and may allow for further increases in REE when used in combination of other stimulant-based ingredients [2].

There is some concern that stimulant-based thermogenic supplements may adversely affect hemodynamic variables, such as heart rate (HR) and blood pressure (BP). Some trials have shown acute increases in HR and BP following ingestion of thermogenic supplements containing caffeine plus ephedra [26, 27]. Others have reported similar elevations in HR and BP following ingestion of a thermogenic product, even when ephedra is not present [1]. Of the ingredients found in the thermogenic product currently being investigated, there is some support suggesting caffeine and GTE can significantly increase energy expenditure without adversely affecting hemodynamic variables [8, 17, 28, 29].

While it is known that these ingredients can have a positive influence on RMR, the magnitude can vary based on the active ingredient and/or combination of active ingredients. Additionally, further safety evaluation is needed on multi-ingredient supplements that contain caffeine because of its potential effect on HR and BP. Therefore, the primary objective for this study was to determine the effects of a thermogenic dietary supplement on RMR in female participants. A secondary objective of this study was to determine the effects of the thermogenic dietary supplement on resting HR and BP.

## Methods

### Participants

Thirteen healthy females (age: 26 ± 11 years; height: 163 ± 9 cm; bodyweight: 64 ± 8 kg; BMI: 24 ± 5) between the ages of 18 and 50 years volunteered to participate in this randomized, double-blind, placebo controlled, cross-over study. All participants reported engaging in resistance exercise and endurance exercise most of days of the week. The research protocol was approved by the University of South Florida Institutional Review Board. Following an explanation of all risks and benefits associated with the experimental protocol, each participant gave her informed consent to participate in this study. Participants were screened for participation based on established criteria set forth by the American College of Sports Medicine [30]. In order to participate in the study, participants needed to be non-smokers, and free from cardiovascular, pulmonary, and metabolic disease. Participants that were categorized as ‘high risk’ for cardiovascular disease according to the American College of Sports Medicine’s risk stratification were excluded from participation in the study. Participants were also excluded as a result of any intolerance or known allergy to the supplement ingredients.

### Experimental design

The study utilized a randomized, double-blind, placebo-controlled, cross-over design. Participants reported to the Performance and Physique Enhancement Laboratory following an overnight fast (a minimum of an 8-hour fast) and a 24-hour avoidance of exercise on two occasions separated by at least 24 h. The laboratory was climate controlled with the average temperature, humidity, and barometric pressure over all testing sessions being 21.4 °C, 47 %, and 762 mmHg, respectively. After arriving to the laboratory, a coin was flipped to randomly determine the order of the dietary supplement ingestion. If the participant were randomized to ingest the thermogenic dietary supplement on the first testing session, they would ingest the alternate treatment (placebo) on the second and final testing session. Likewise, if the participant were randomized to ingest the placebo treatment on the first testing session, they would ingest the alternate treatment on the subsequent laboratory visit. Testing sessions for both laboratory visits occurred between the hours of 5:30 am and 9:30 am, with the majority of all assessments beginning at 7 am.

### Testing sessions

Upon arriving to the laboratory, participants were encouraged to use the restroom to empty their bladders. Next, the participant had their body weight measured on a physician beam scale (Health-O-Meter, Model 402KL, McCook, IL, USA) and then sat in a reclined position with their feet elevated for a 5-minute period. After sitting quietly for 5 min, participants had their resting heart rate and blood pressure recorded using an automated, oscillometric blood pressure monitor (Omron 5 series Model BP742, Lake Forest, IL, USA). This method of automated, oscillometric blood pressure measurement has been validated in the scientific literature [31]. Heart rate and blood pressure were measured in triplicate and the average of the three readings was recorded.

Next, the participant remained in a reclined position for an additional 5 min prior to the resting metabolic rate (RMR) measures. All RMR measures were made using a Cosmed FitMate Pro™ (Cosmed, Italy). The FitMate Pro™ contains a turbine flow meter for measuring ventilation and a galvanic fuel cell oxygen sensor for analyzing the fraction of oxygen in the expired gases. To sample the expired air, a facemask was placed over the participant’s face and attached to the turbine flow meter. The device uses standard metabolic formulas to calculate oxygen uptake, and energy expenditure is calculated using a fixed respiratory quotient (RQ) of 0.85. The FitMate Pro™ also conducts a self-calibration before each measurement, and at two other times (5 and 10 min) during the 15-minute RMR assessment. The device has been validated with a Douglas bag for non-obese and obese subjects and was found to calculate RMR accurately (*r* = 0.97, *p* = 0.579) [32]. Intra and inter-day test-retest correlation calculated for the device used in the present study were as follows: intra-day RMR Pearson correlation was *r* = 0.96 (*p* < 0.01) and the inter-day RMR Pearson correlation was *r* = 0.90 (*p* < 0.01). Intra-day RMR ICC was 0.981 and the inter-day RMR ICC was 0.946.

At baseline, two consecutive RMR tests were conducted and the lower of the two measured RMR values was recorded as the baseline RMR value. During the RMR test, the participant was instructed to relax, breathe normally, and remain as still as possible for the duration of the 15-minute test. The first 5 min of data collection was discarded [33] and the final 10 min of data collected was used in the calculation of the resting metabolic rate.

**Fig. 1:**
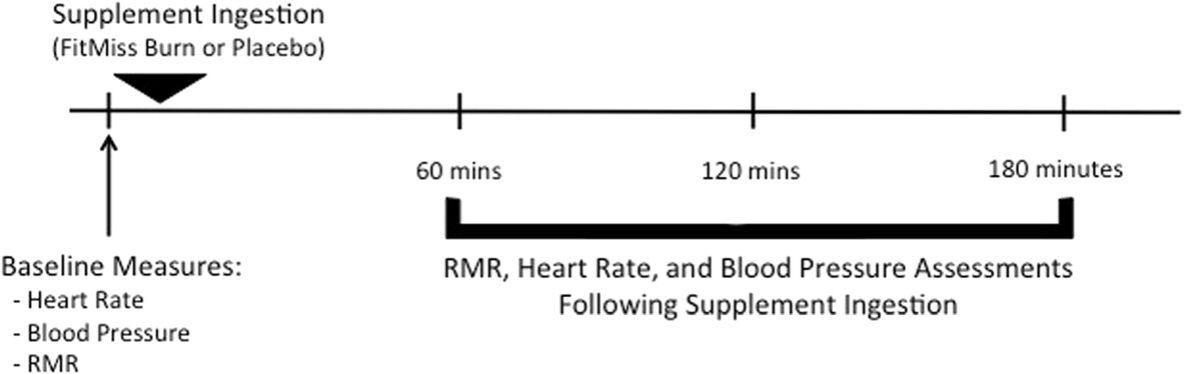
Overview of Testing Sessions

After baseline RMR was established, the participant ingested two capsules of either the thermogenic dietary supplement or placebo treatment with 8 ounces of cold water. After ingestion of the supplement treatments, three more heart rate, blood pressure, and RMR assessments were made at 1-hour, 2-hours, and 3-hours post ingestion. Figure 1 presents an overview of the study test sessions.

### Supplement

The thermogenic dietary supplement treatment and placebo were ingested in capsule form, and two capsules were ingested per dose. Capsules were identical in appearance and taste. The ingredients in the thermogenic dietary supplement treatment (commercially available as FitMiss Burn™) are presented in Table 1, while the placebo contained only inert ingredients (650 mg of maltodextrin and 88.8 mg of hemp protein). Following the completion of two baseline RMR tests, the participant ingested two capsules of the thermogenic supplement or the placebo treatment with eight ounces of water. Supplement ingestion was witnessed by research personnel for all testing sessions. There were no intolerances or allergic reactions to the supplements reported during the course of the study.

**Table 1:** Supplement facts panel

Supplement Facts		
Serving Size: 2 capsules		
Serving Per Container: 45		
Amount Per serving		% Dv^a^
Vitamin B3 (as Niacinamide)	2 mg	10 %
Vitamin B5 (as Calcium D-Pantothenate)	2 mg	20 %
Magnesium (as Magnesium Gluconate)	3 mg	1 %
Zinc (as Zinc Monomethionine)	666 mcg	4 %
Chromium (as Chromium Polynicotinate)	66 mcg	55 %
Potassium (as Potassium Aspartate)	1.16 mg	0 %
**Revolutionary Women’s 6 Stage Weight Loss Blend**	1,499 mg	^b^
**Stage 1&2: Energy And Fat Metabolizer**		
Green Tea Extract (40 % EGCG)(*Camellia Sinensis*)(Leaf), Caffeine Anhydrous (150 mg), Guarana Seed Extract (*Paullinia Cupana*)(22 % Caffeine), Yerba Mate (*IIex Paraguariensis*)(Leaf),Vinpocetine (*Crioceras Longiflorus*) Whole Plant Extract		
**Stage 3: Appetite Balancing Weight Management Control**		
Glucomannan (*Amorphophallus Konjac*), White Kidney Bean (*Phaseolous Vulgaris*), Raspberry Ketones, Alpha Lipoic Acid, Cayenne 40,000 HU/g (*Capsicum Annum*)(Pepper), Chromium(Chelate)		
**Stage 4: Anti-Stress Mood Balancer**		
DL-Phenylalanine, 5-HTP (*Griffonia Simplicifolia*)(Seed), Gingko Biloba Leaf Extract, Turmeric(*Curcuma Longa*) Root, Garlic (*Allium Sativum*), *Echinacea Augustifolia* Root		
**Stage 5: Water Shedding Diuretic Complex**		
Uva Ursi (*Arctostaphylos Uva-Ursi*)(Leaf)(Contains Arbutin, Methyl-Arbutin), Dandelion (*Taraxacum Officinale*)(Root) Extract 20:1 (Contains Taraxol & Taraxerol), Potassium Aspartate		
**Stage 6: Digestive Enzyme Aid**		
DigeSEB® (Amylase, Protease Blend [I,II,III], Lipase, Lactase, HemiSEB® Cellulase, Maltase,Invertase, Bromelain, Peptizyme SP® Papain and Alpha-Galactosidase), Almond (*Prunus Amygdalus*) Oil Powder, Corn (*Zea Mays*) (Silk), Kelp (Ascophyllum Nodosum), Apple (*Malus Domesticus*) Pectin		

^a^Percent Daily Values are based on a 2,000 calorie diet

^b^Daily value not established

Other Ingredients: Hypromellose, Magnesium Stearate, Microcrystalline Cellulose

### Statistical analysis

Statistical analyses of the data were analyzed via a 2-factor [2x4] within-subjects repeated measures analysis of variance (ANOVA) using SPSS version 22.0. Post-hoc tests were analyzed via paired samples t-tests. In addition, baseline RMR data for the two separate testing days was compared via a paired samples *t*-test. Incremental area under the curve (AUC) was calculated for each treatment (thermogenic supplement and placebo) using the trapezoidal method as described by Brouns et al. [34]. A paired samples *t*-test was used to determine AUC differences between the two treatments. A criterion α-level of p ≤ 0.05 was used to determine statistical significance.

## Results

### Resting metabolic rate

**Fig. 2:**
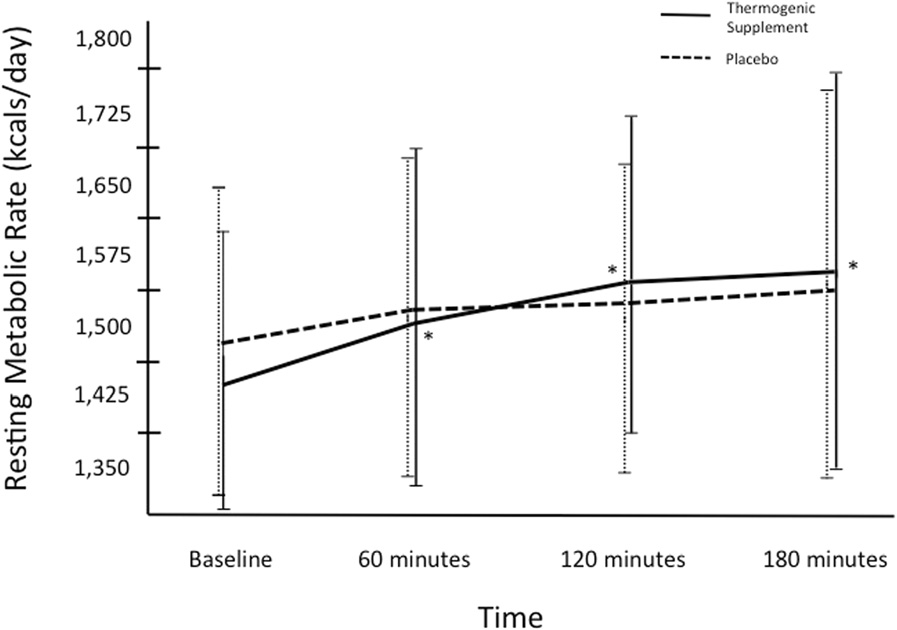
Change in resting metabolic rate (RMR) from baseline to 3-hours post ingestion. Data is expressed as Mean SD. * = significant increase in RMR as compared to baseline for thermogenic supplement treatment (*p* < 0.05)

**Fig. 3:**
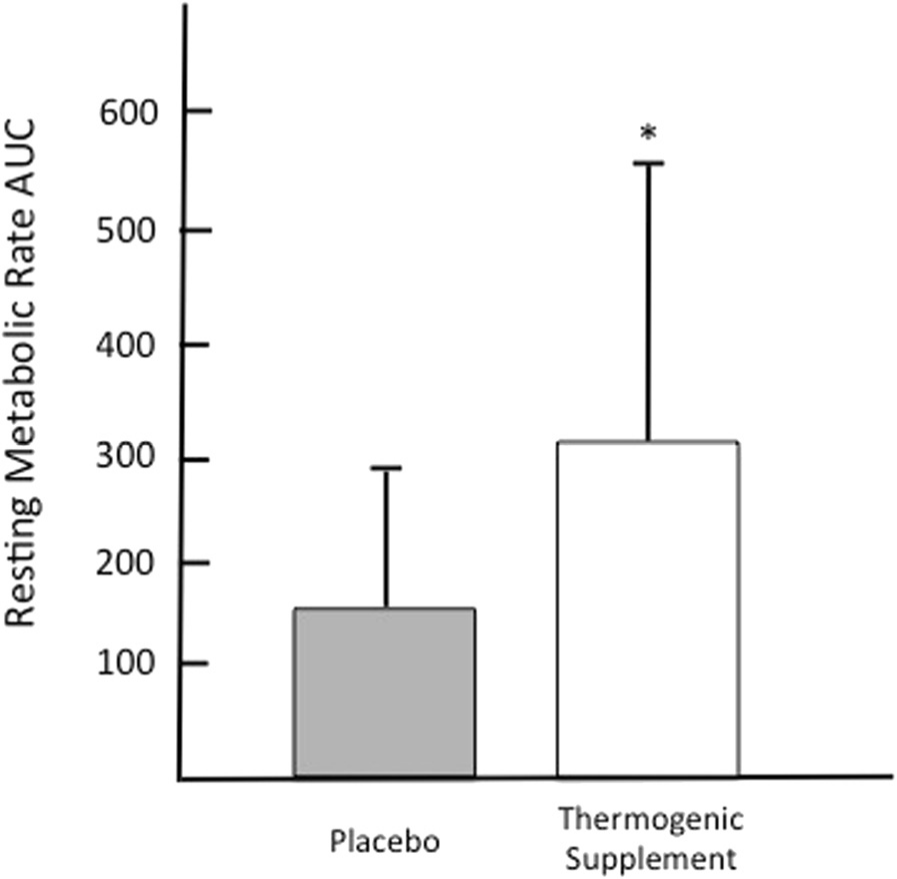
Baseline Subtracted Area Under the Curve (kcals/3 hours). * = significant difference between treatments (*p* < 0.05)

Regarding the normality of the RMR measures analyzed in this study, the standardized skewness and kurtosis coefficients were all within the range of ± 1.0; therefore, the RMR data were determined to be normally distributed [35]. The coefficient of variation for RMR at baseline was 7.0 and 8.4 % for the treatment and placebo treatments, respectively. Paired samples *t*-test revealed no significant difference in baseline RMR between the two treatments. Repeated measures ANOVA revealed a significant treatment x time (*p* = 0.045, F = 2.97) and main effect for time (*p* = 0.001, F = 6.59) relative to raw RMR data, but no difference in the main effect for treatment (*p* = 0.895, F = 0.18) was observed. Post-hoc analysis revealed that the thermogenic dietary supplement treatment significantly increased RMR at 60 min, 120 min, and 180 min post ingestion (*p* = 0.025, 0.008, 0.008; effect size = 0.41, 0.63, and 0.63, respectively), as compared to baseline RMR values (Fig. 2). No significant changes in RMR were observed for the placebo treatment in comparison with baseline values. Specifically, RMR was increased by 5.6, 8.5, and 9 % in the thermogenic supplement, while the placebo treatment increased RMR by 2.7, 2.8, and 4.7 % above baseline at 60, 120, and 180-min post ingestion, respectively. Relative to AUC comparisons, a significant difference (*p* = 0.016, t = 2.82) was observed between the thermogenic supplement and placebo treatments (Fig. 3).

### Hemodynamic response

Heart rate was not significantly affected at any time point with either supplement (*p* > 0.05). For systolic blood pressure (SBP), no treatment x time interaction effect (*p* = 0.748) was observed, but a main effect for treatment (*p* = 0.031) and main effect for time (*p* = 0.005) was revealed. For diastolic blood pressure (DBP), a treatment x time interaction effect (*p* = 0.028), a main effect for treatment (*p* = 0.005), and a main effect for time (*p* = 0.009) were observed. Raw data for RMR and hemodynamic variables is summarized in Table 2.

**Table 2:** Resting metabolic rate and hemodynamic summary data

	Baseline		60 minutes		120 minutes		180 minutes	
	TS	Plac	TS	Plac	TS	Plac	TS	Plac
Resting Metabolic Rate (kcals/day)	1,435 (204)	1,476 (176)	1,515* (190)	1,516 (161)	1,557* (182)	1,518 (171)	1,564* (207)	1,546 (202)
Heart Rate (beats/min)	57.2 (9)	59.8 (9)	56.9 (10)	60.0 (10)	56.8 (6)	58.6 (9)	56.9 (8)	60.2 (12)
Systolic Blood Pressure (mmHg)	113 (15)	110 (15)	120 (17)*	114 (18)	117 (13)	113 (2)	120 (15)*^#^	116 (16)*^#^
Diastolic Blood Pressure (mmHg)	65 (10)	65 (10)	74 (10)*^#^	68 (9)*^#^	75 (14)*^#^	66 (11)^#^	74 (11)*	70 (12)*

TS = thermogenic supplement. Plac = placebo. Data is presented as mean (± standard deviation). * = p < 0.05 within group change as compared to baseline value. ^#^ = p < 0.05 between groups difference at same time point

## Discussion

The purpose of this study was to examine the effects of a multi-ingredient thermogenic supplement on RMR and hemodynamic function in healthy females. The results of the study show that acute ingestion of the thermogenic dietary supplement, which contains caffeine, GTE, guarana seed extract, chromium polynicotinate, and yerba mate leads to a significant increase in RMR in young, healthy women, without adversely affecting HR or BP.

Thermogenic supplements are marketed to the general public to enhance fat loss via an increase in energy expenditure. In the current study, the dietary supplement treatment experienced greater elevations in RMR values compared to baseline, whereas the placebo treatment did not. Previous research has demonstrated that caffeine, in combination with other herbal ingredients, can increase RMR for up to three hours following ingestion [1, 2, 9, 29]. In agreement with these findings, the current study demonstrated a 9 % increase in RMR three hours post-ingestion of the thermogenic dietary supplement treatment, whereby RMR in the placebo treatment increased 4.7 %. Although earlier thermogenic supplement studies have shown significant increases in RMR ranging from 10-29 % above baseline [1, 12, 36], differences could be attributed to the dosages used, the combination of ingredients, and the concentrations of individual ingredients.

Caffeine is a common ingredient found in most thermogenic supplements due to its effects on energy expenditure. Caffeine alone [8, 37], caffeine plus GTE [7, 37, 38], and caffeine in thermogenic supplements containing other herbal ingredients [2] have been shown to induce greater energy expenditure (when compared to a placebo), which could impact weight loss over time if consumed chronically. Hoffman et al. [1] reported a 17.9 % increase in RMR for female participants following ingestion of a coffee beverage containing additional caffeine, green tea extract, niacin, and garcinia cambogia. Supporting these findings, Wilborn et al. [29] reported a 15.5 % increase in RMR three hours post-ingestion of a thermogenic product containing caffeine, GTE, and yohimbine-HCl. Furthermore, a study conducted by Dalbo et al. [36] demonstrated a 10.5 % increase in RMR over a three hour period following ingestion of a thermogenic drink containing caffeine and EGCG. The results of the current study showed a 9 % increase in RMR following ingestion of the thermogenic dietary supplement. While this value is lower than that of previous studies examining the effects of a caffeine-containing thermogenic on RMR, the dose of caffeine in the current product (150 mg) was lower than that reported in previous studies (200-400 mg). Therefore, it is logical to conclude that the differences observed in RMR is likely due to the differences in caffeine dosage, and possibly the combination of other ingredients.

While GTE alone has been shown to increase RMR, the combination of GTE and caffeine can significantly increase catecholamine release, leading to further increases in RMR. [1] Therefore, the increase in RMR observed in the current study is likely the result of the combination of caffeine and GTE. Although garcinia cambogia and chromium polynicotinate have some support to suggest they can acutely enhance energy expenditure following ingestion [12], the relatively small amounts found in the current product are likely not at levels high enough to affect RMR. Additionally, the research on these ingredients increasing metabolism in humans is inconclusive [39, 40].

Yerba mate has been shown to suppress appetite and prevent diet-induced obesity in rats [41]. Additionally, when combined with caffeine, the synergistic effect may enhance weight loss. Although there is some discussion that yerba mate may assist in weight loss, currently its role in increasing energy expenditure is not well understood and may be negligible.

The secondary purpose of this study was to examine the effects of the thermogenic dietary supplement on resting hemodynamic variables. Acute ingestion of the thermogenic dietary supplement did not significantly alter heart rate but changes in both systolic and diastolic blood pressure were observed. While long-term consumption of caffeine has minimal effect on hemodynamic function [8, 42, 43], there are some studies that have demonstrated an acute increase in SBP and HR following ingestion of a thermogenic supplement [1, 26]. Similar to previous studies, the present study observed a significant increase in BP across time, with both treatments observing a slight elevation in SBP values over the three-hour testing period. Furthermore, DBP increased over time for both treatments; however, ingestion of the thermogenic dietary supplement caused a significantly greater increase in DBP compared to the placebo treatment. It should be noted that although changes in DBP were significant, all values stayed within normal clinical ranges (<80 mmHg). Given that SBP reached 120 mmHg at the 3-hour post-ingestion time point, this value meets the threshold for pre-hypertension (ranging from 12 to 139 mmHg). In consideration of this finding, supplementing with the thermogenic dietary supplement used in the current investigation may be ill-advised for those with increased risk for hypertension or prehypertension.

There were several limitations of the present study. One limitation was the lack of control for menstrual phase. However, even though menstrual phase was not controlled for, the findings of the study indicate that one can expect an acute (3-hour) increase in RMR, regardless of the timing of the menstrual cycle. Other limitations include a relatively small sample size and the lack of a CO_2_ sensor, which precluded the observation of substrate utilization. Also, this study employed no survey of the typical caffeine intake of the participants. Further, no information was collected that could have identified the participants as caffeine naïve or habitual caffeine users.

## Conclusion

When the participants ingested the thermogenic dietary supplement treatment, they experienced greater elevations in RMR values as compared to baseline, whereas no elevations in RMR were observed following the placebo treatment. These elevations came with no adverse effects relative to resting heart rate and diastolic blood pressure values, but due to the elevations in systolic blood pressure, caution should be taken for those with increased risk for hypertension or pre-hypertension. Taken on a daily basis, FitMiss Burn™ supplementation may increase overall energy expenditure, possibly leading to reductions in fat mass over time. Future work should investigate the effectiveness and safety of ingesting the dietary supplement over a longer period of time (several months) to determine if reductions in fat mass are observed.
